# The successes and pitfalls: Deep‐learning effectiveness in a Chernobyl field camera trap application

**DOI:** 10.1002/ece3.10454

**Published:** 2023-09-05

**Authors:** Rachel E. Maile, Matthew T. Duggan, Timothy A. Mousseau

**Affiliations:** ^1^ Department of Biological Sciences University of South Carolina Columbia South Carolina USA; ^2^ K. Lisa Yang Center for Conservation Bioacoustics, Cornell Lab of Ornithology Cornell University Ithaca New York USA; ^3^ Department of Natural Resources and the Environment Cornell University Ithaca New York USA

**Keywords:** animal abundance and occupancy, artificial intelligence, camera trap imagery, Chernobyl, convolutional neural network, machine learning

## Abstract

Camera traps have become in situ sensors for collecting information on animal abundance and occupancy estimates. When deployed over a large landscape, camera traps have become ideal for measuring the health of ecosystems, particularly in unstable habitats where it can be dangerous or even impossible to observe using conventional methods. However, manual processing of imagery is extremely time and labor intensive. Because of the associated expense, many studies have started to employ machine‐learning tools, such as convolutional neural networks (CNNs). One drawback for the majority of networks is that a large number of images (millions) are necessary to devise an effective identification or classification model. This study examines specific factors pertinent to camera trap placement in the field that may influence the accuracy metrics of a deep‐learning model that has been trained with a small set of images. False negatives and false positives may occur due to a variety of environmental factors that make it difficult for even a human observer to classify, including local weather patterns and daylight. We transfer‐trained a CNN to detect 16 different object classes (14 animal species, humans, and fires) across 9576 images taken from camera traps placed in the Chernobyl Exclusion Zone. After analyzing wind speed, cloud cover, temperature, image contrast, and precipitation, there was not a significant correlation between CNN success and ambient conditions. However, a possible positive relationship between temperature and CNN success was noted. Furthermore, we found that the model was more successful when images were taken during the day as well as when precipitation was not present. This study suggests that while qualitative site‐specific factors may confuse quantitative classification algorithms such as CNNs, training with a dynamic training set can account for ambient conditions so that they do not have a significant impact on CNN success.

## Graphical abstract

Convolutional neural networks (CNNs) are an increasingly popular tool for classifying camera trap imagery. This study observed the effects of ambient factors, such as outdoor meteorology and degree of daylight, on CNN accuracy.

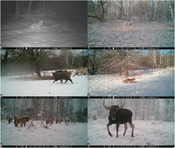

## 1 INTRODUCTION

Although camera traps (i.e., motion activated cameras) have been used for decades as a means of observing animal species in a wide variety of habitats while causing minimal disturbance (O'Connell et al., 2011), it is only recently that they have become cost effective for widespread deployment in the field. Camera traps have been widely used to observe various aspects of populations such as animal density and abundance (O'Brien et al., 2003; Rowcliffe et al., 2008). Arguably, camera trap studies have become the most appropriate means of obtaining occupancy and abundance data in most environments, even in difficult terrain or habitats with restricted human access (Karanth, 1995; Schlichting et al., 2020). Furthermore, camera trap observations of important species can serve as a basis for estimating the overall ecological health of an ecosystem (Karanth, 1995).

However, in order to most effectively estimate animal distribution and abundance, numerous camera traps must be deployed with a high sampling effort (Di Bitetti et al., 2006). As a consequence of a large number of camera traps in a singular or multiple studies, an expansive number of images need to be filtered and labeled. Conventionally, this requires a huge amount of human labor to classify species within each image, often through the use of citizen scientists (Swanson et al., 2015; Willi et al., 2019). Furthermore, outdoor meteorology has been shown to influence camera trap effectiveness, such as detection distance shortening during rainy weather because of moisture reducing the contrast between an animal and its background (Kays et al., 2010).

Due to the considerable time and effort expended by researchers when classifying camera trap images, many studies have deployed the use of machine learning to rapidly classify animal species and anthropogenic objects, including humans and vehicles (Duggan et al., 2021; Tabak et al., 2018). In fact, some studies have even found that machine‐learning models can sometimes outperform the average citizen scientist with regard to accuracy (Norouzzadeh et al., 2018; Whytock et al., 2021). One of the most popular machine‐learning architectures are CNNs, which are deep‐learning algorithms that have a variety of branching methodologies in their construction, such as recurrent convolutional neural networks, to suit a variety of problems within the scope of ecology (O'Shea & Nash, 2015). Overall, CNNs are now widely used in camera trap studies for the purposes of image recognition and classification (Gomez Villa et al., 2017). Furthermore, CNNs have the potential to save researchers an extensive amount of time, and thus human labor can be redirected toward other scientific purposes (Norouzzadeh et al., 2018; Swanson et al., 2015).

The majority of machine‐learning architectures require an exhaustive amount of images to train such animal detectors and classification algorithms, thus being a significant upfront cost to construct such deep‐learning models. Transfer learning is a machine‐learning method that recycles a preconstructed neural network, typically trained on an extensive dataset, by only adjusting the final steps of say a CNN architecture. The utilization of transfer learning is rapidly enabling researchers to use a relatively small training image set, yet still correctly classifying animals (Duggan et al., 2021; Hu et al., 2015; Schneider et al., 2020; Shao et al., 2015). Through the use of transfer learning, CNN performance can be fine‐tuned and improved for more specific classes or objects of interest (Yosinski et al., 2014). While a smaller amount of images can be used to make a satisfactory classifier, such models should always require out‐of‐sample images to validate such model constructions to a study's general dataset (Tabak et al., 2020).

While transfer learning and other methods, such as data augmentation, are showing promise in reducing the effort to train models for animal occupancy models, these models can be improved by adding a wider array of images. A wide variety of unique images are necessary to train these models due to external factors at camera sites, yet few studies mention their effects. High false positive rates have been reported due to dynamic images with background clutter, or variations, such as shadows and swaying vegetation due to wind (Newey et al., 2015; Zhang et al., 2016). False positives due to the ambient environment can occur due to thermal heterogeneity, in which surrounding vegetation triggers camera traps due to it being a different temperature than the background (Welbourne et al., 2016). With external variables, such as degree of light, affecting aspects of image quality and object contours, CNN accuracy may in turn be affected as the model has difficulty in distinguishing animal species from the background in which they inhabit. Furthermore, effectiveness at the camera trap level can be affected by the target species and camera quality. Here, we examine the effects of meteorological conditions and daylight levels on CNN accuracy and provide recommendations for building the training dataset used by a CNN for evaluating a uniquely trained model for the classification of terrestrial animals.

## 2 METHODS

### 2.1 Study site

The nuclear accident at Chernobyl, Ukraine (51′27″63° N, 30′22″19° E) occurred in 1986 and released around 1 × 10^19^ Bq of radioactivity that was transported over long distances across the northern hemisphere but especially throughout eastern Europe and Scandinavia (Evangeliou et al., 2016). The highest levels of contamination are found within the Chernobyl Exclusion Zone (CEZ) of Ukraine, which consists of 2600 square kilometers surrounding the plant. The local habitat consists of thick forests and fallow agricultural lands which have been closed to the public due to high levels of radiation. Thus, Chernobyl offers the unique opportunity to explore the ecological effects of radiation, as well as terrestrial wildlife without human interference (Mousseau, 2021; Mousseau & Møller, 2014). Due to restricted human access, dangerous levels of radiation, and now a war‐stricken environment caused by Russian aggression, camera traps are ideal for observing animal species in Chernobyl safely (Schlichting et al., 2020).

### 2.2 Camera trap sampling design

**FIGURE 1:**
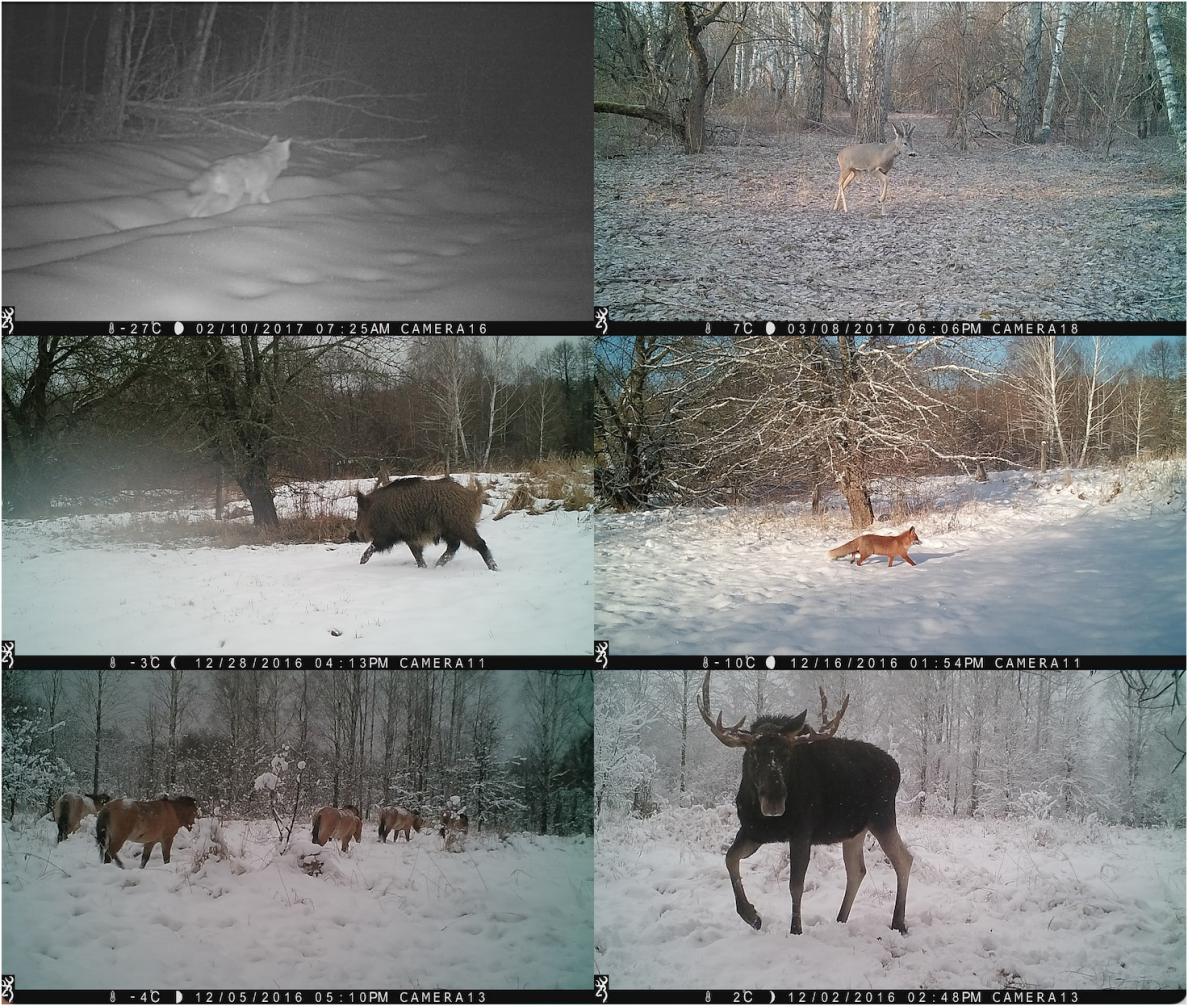
Sample photographs taken from camera traps in Chernobyl. Starting from top left and proceeding clockwise, species are the following: gray wolf (*Canis lupus*), roe deer (*Capreolus capreolus*), red fox (*Vulpes vulpes*), moose (*Alces alces*), Przewalski's horse (*Equus ferus*), and boar (*Sus scrofa*).

**FIGURE 2:**
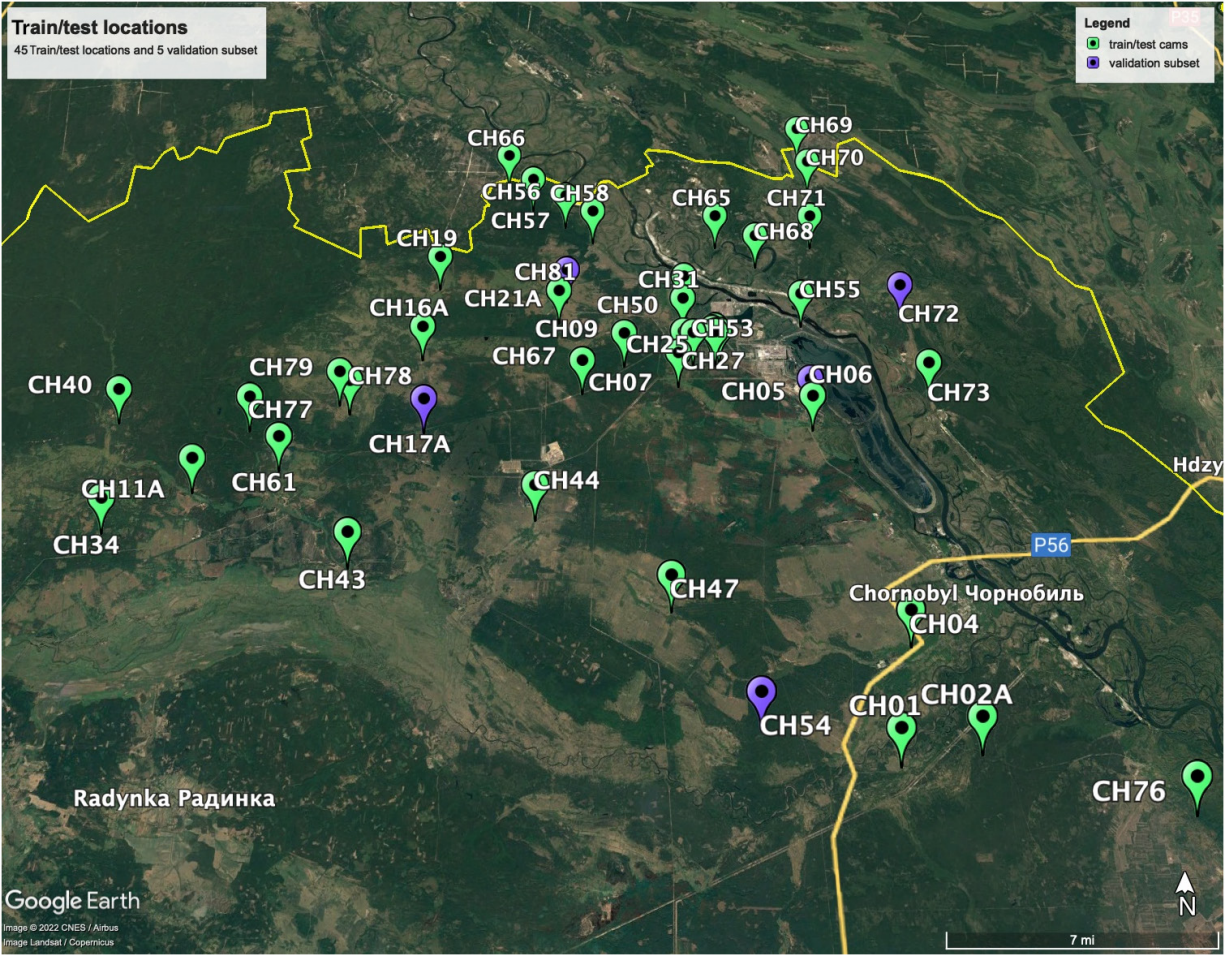
Map of 45 camera locations within the Chernobyl Exclusion Zone whose images were used to create the model. All cameras shown were part of the train/test set, with a validation subset shown in purple. Five cameras are labeled with an “A” to show that their location was also used in the case study but from a different time range Google Earth Pro 7.3.6.9285 (2022).

We observed 14 animal species in camera trap images taken from 45 locations across the CEZ We used relatively inexpensive consumer‐grade Browning Recon Force FHD trail cameras for this study. Trail cameras were placed about 1.22 m above the ground and were generally oriented toward the north so as to avoid glare (see Figure 2.2 in Appendix S2). These cameras use passive infrared detectors (PIR) to sense motion and a series of eight still images were recorded when an animal was detected. Because of their sensitivity, the traps are generally nondiscriminatory with respect to the species they capture—from moose that have a height up to three meters to weasels that only weigh a few ounces. We employed opportunistic sampling design, that is to say, we placed cameras in areas that were accessible and had a high likelihood of animals passing through ‐ such as clearings. Our sampling unit consisted of individual camera stations. These cameras have a reported detection distance of 16.76 m, a trigger speed of 0.67 s. They were programmed to capture images at a 10 MP resolution. Representative images are shown in Figure 1. Camera trap images used to train and validate the model were taken between the months of November 2019 and May 2020. The traps were placed throughout the CEZ in a variety of locations, including wooded areas and fields (see Figure 2). Camera traps were deployed within an approximately 1500 square kilometer area in the CEZ (for a density of 1 camera per 33.33 km) at elevation ranges from 300 to 500 feet. This area consists of a humid continental climate with warm summers and snowy, cold winters.

### 2.3 Convolutional neural network development

**FIGURE 3:**
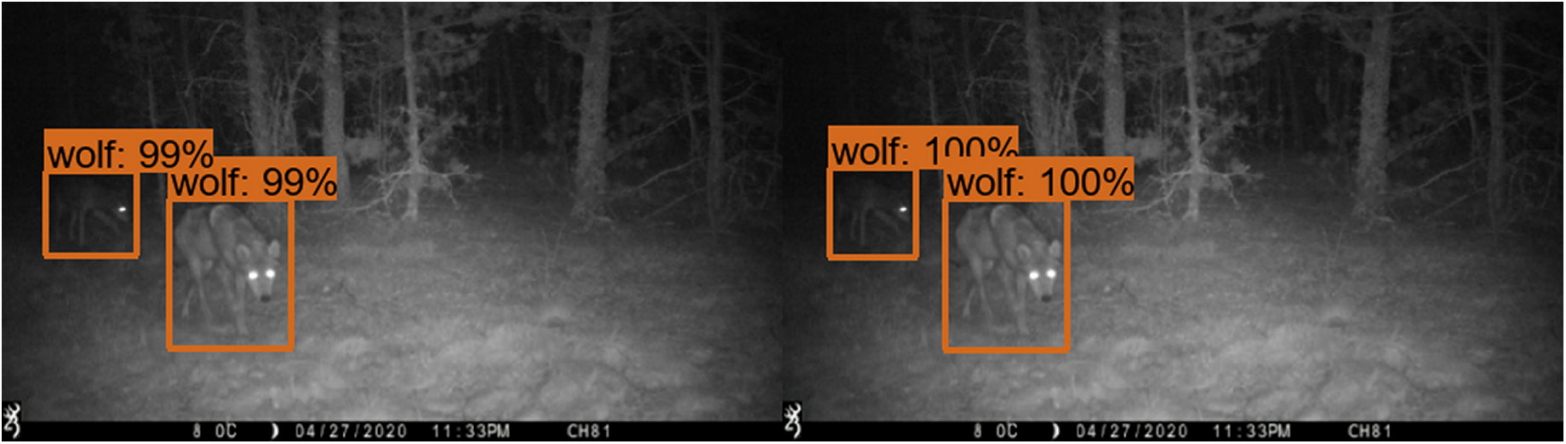
Bounding boxes with confidence predictions around a target object. Computer‐generated bounding boxes are shown on the left side and human‐labeled bounding boxes are shown on the right side.

Following an application of Duggan et al. (2021) for our Chernobyl study site, we explored the consequences of utilizing fewer images and the factors necessary to consider when implementing CNN architectures in field camera trap imagery. A premade extension of the CNN, Faster‐RCNN, was trained with this relatively small image set in order to reduce the running time of the model and to enhance computational efficiency (Ren et al., 2017; Schneider et al., 2018). Emphasis was placed on including images in the training dataset that showed animals in a wide variety of positions and motions so as to give the model multiple perspectives of a species. Using the graphical image annotation tool LabelImg (Tzutalin, 2015), we drew bounding boxes with a label around each species to establish ground truths, which consisted of the correct, or real, classification of each object (see Figure 3). These bounding boxes distinguish the object from background noise. If necessary, multiple bounding boxes per image were labeled to account for multiple animals. Overlapping bounding boxes were allowed in the instance that animals were superimposed in the image. Furthermore, if only part of an animal was present in an image, such as a foot or a tail, these were also labeled with their corresponding species. The defining bounding box was transferred to a CSV format with the training processes utilized in the Tensorflow training framework (Abadi et al., 2015).

### 2.4 Train/test sets

Before classification, all images were resized to 1920 × 1080 pixels that is typical of camera trap studies so as to increase processing speed and improve efficiency of limited computational resources. Using the widely accepted 90/10 split (Fink et al., 2019), 90% of images were divided into a training subset and 10% were divided into a testing subset. Only images that displayed a unique perspective of each species were included in the training dataset so as to enhance model training. We took a stratified random sample across 45 cameras and held certain cameras out from evaluation that had significant vegetation triggering. In the conditional sampling of images, a range of meteorological conditions and light levels were included. In total, 4022 images acquired from 45 cameras placed across the CEZ were classified, with 3620 images in the training dataset and 402 images in the testing dataset.

### 2.5 Model validation

A validation subset was created by classifying images from five cameras with high species diversity from throughout the study site. We applied the trained model to this validation subset to extrapolate the trained CNN to a different set of images. Therefore, the validation dataset is separate from the train and test sets. A total of 8135 images were used in this subset, including 2610 true negatives. Images from this set were also labeled with LabelImg to evaluate model performance metrics. We ran the trained model at a confidence threshold of 0.9 on these images to evaluate model performance. Validation metrics were compared to the train/test metrics to ensure that the model was not overfitted.

### 2.6 Case study

**FIGURE 4:**
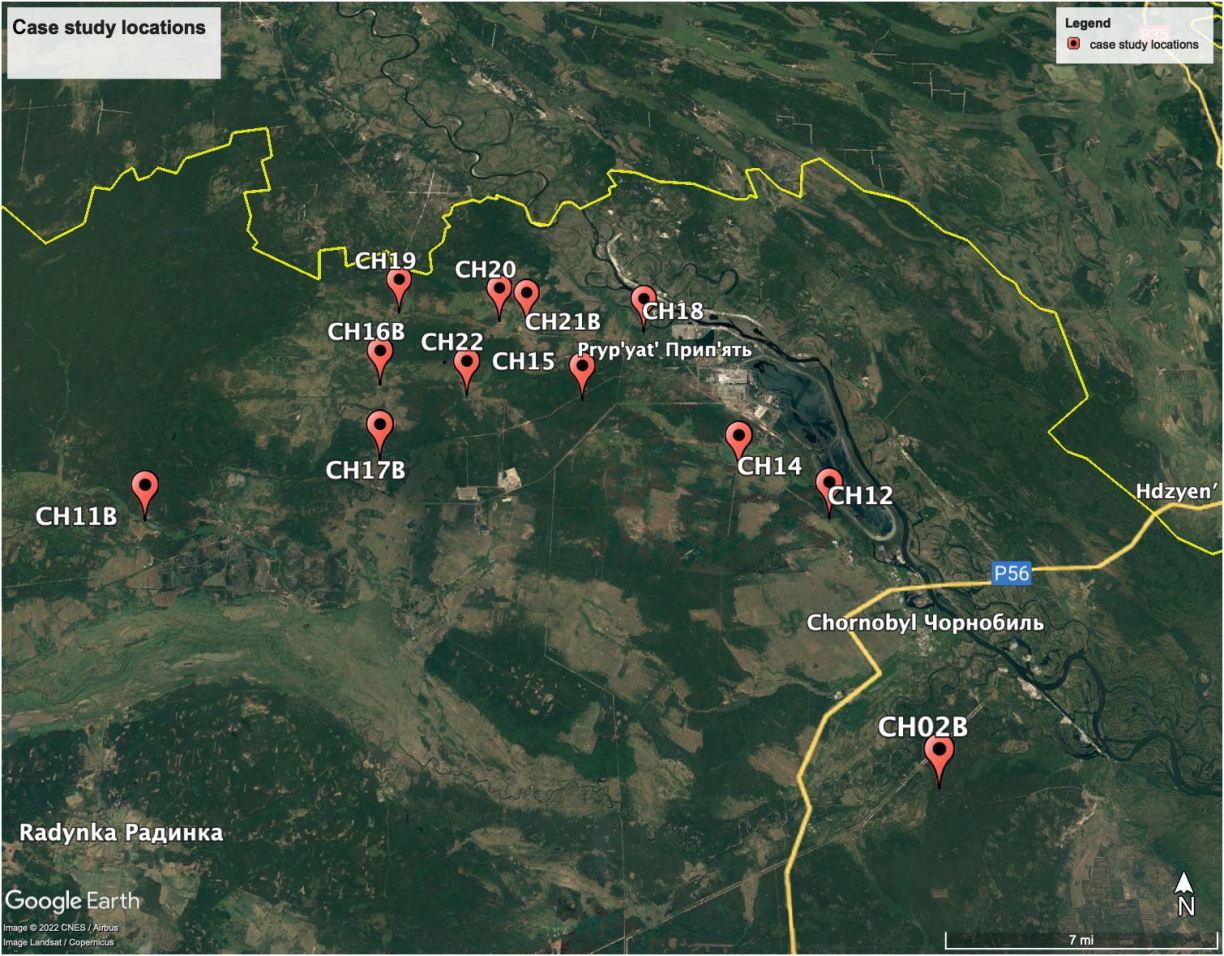
Map of 12 camera locations within CEZ that were used as part of the case study. Five cameras are labeled with a “B” to show that their location was also used in the train/test set but from a different time range Google Earth Pro 7.3.6.9285 (2022).

Once the model was effectively trained and validated, it was applied to 9576 images from 12 randomly selected cameras not included in the training, test, or validation dataset within the Chernobyl Exclusion Zone (see Figure 4). These 12 cameras contained images not included in the 45‐camera training/testing set because of the chance of labeled data interfering with the unsupervised images. In other words, in order to determine how the model would perform on a random selection of data, we chose separate camera traps from our training camera traps. The images from these camera traps within the case study are different from images in the train, test, and validation sets. These cameras were all generally placed in clearings within wooded areas with little variation in the surrounding habitats, with the exception of an occasional dirt road (CH16B's site) or abandoned structures (sites from CH20 and CH21B). These 12 cameras contained images from November 2016 to March 2017. Images showing the site‐specific factors of each of the camera traps can be found in Appendix S2. Data from the nine most common classes (denoted by an asterisk in Table 1) were selected for analysis. A total of 114 unique animal classifications, defined as events, were contained within the 9576 images taken. Unique animal classifications consisted of photographs of an animal, or a group of animals, captured by the camera traps. Therefore, there were 114 events consisting of 182 unique animals. Furthermore, if consecutive images contained members of the same species taken less than 1 h apart, these were classified as a single event to avoid pseudo‐replications. We assumed that camera events only contained one type of species. In more than 2 million images of Chernobyl, only two instances occurred in which multiple different species were present in an image at the same time. A classification of an animal had to be above a critical 90% threshold for taking the model‐assigned accuracy predictions into account. Furthermore, images were clarified to one species with the species occurring most often as the correct classification. For example, if the CNN classified seven of the eight images as a red deer and the last as a roe deer, the event was clarified to consist of a red deer.

**TABLE 1:** Image distribution for test and train subsets for 14 different classes within 4022 images taken from 45 camera trap locations.

Class	Train images	Test images
Badger	157	17
Bird	18	2
Boar*	176	19
Cow	83	9
Fox*	270	30
Hare*	471	52
Horse*	320	35
Lynx	62	7
Moose*	475	53
Raccoon Dog*	154	17
Red Deer*	504	56
Roe Deer*	458	51
Weasel	51	6
Wolf*	243	27

*Note*: The nine most common classes are delineated by an asterisk.

The predicted counts made by the convolutional neural network were compared with the actual counts originally made by human observers. The actual counts were reassessed by a second observer to ensure accuracy. CNNs can be defined by varying levels of success: at the lowest level a success consists of merely separating an animal (no matter the species or count) from vegetation. At a slightly more demanding level, a success can be defined as not only detecting an animal but detecting the correct species. Finally, at the most exacting level, a success can be defined as detecting both the right species and number of animals present in a given image. For the purposes of this research, we chose to use the most stringent parameters for success: events in which the model correctly identified the species and number of animals present in each image were labeled as successes, and all other events were labeled as failures. Due to this definition, the “success rate” of 50.88% is different from the aforementioned accuracy rate of 81.31. Failures consisted of false positives, false negatives, and misclassifications. Our main objective was to attribute failures to a variety of variables, including but not limited to cloud cover, wind speed, temperature, precipitation, and amount of daylight.

### 2.7 Statistical analysis

**FIGURE 5:**
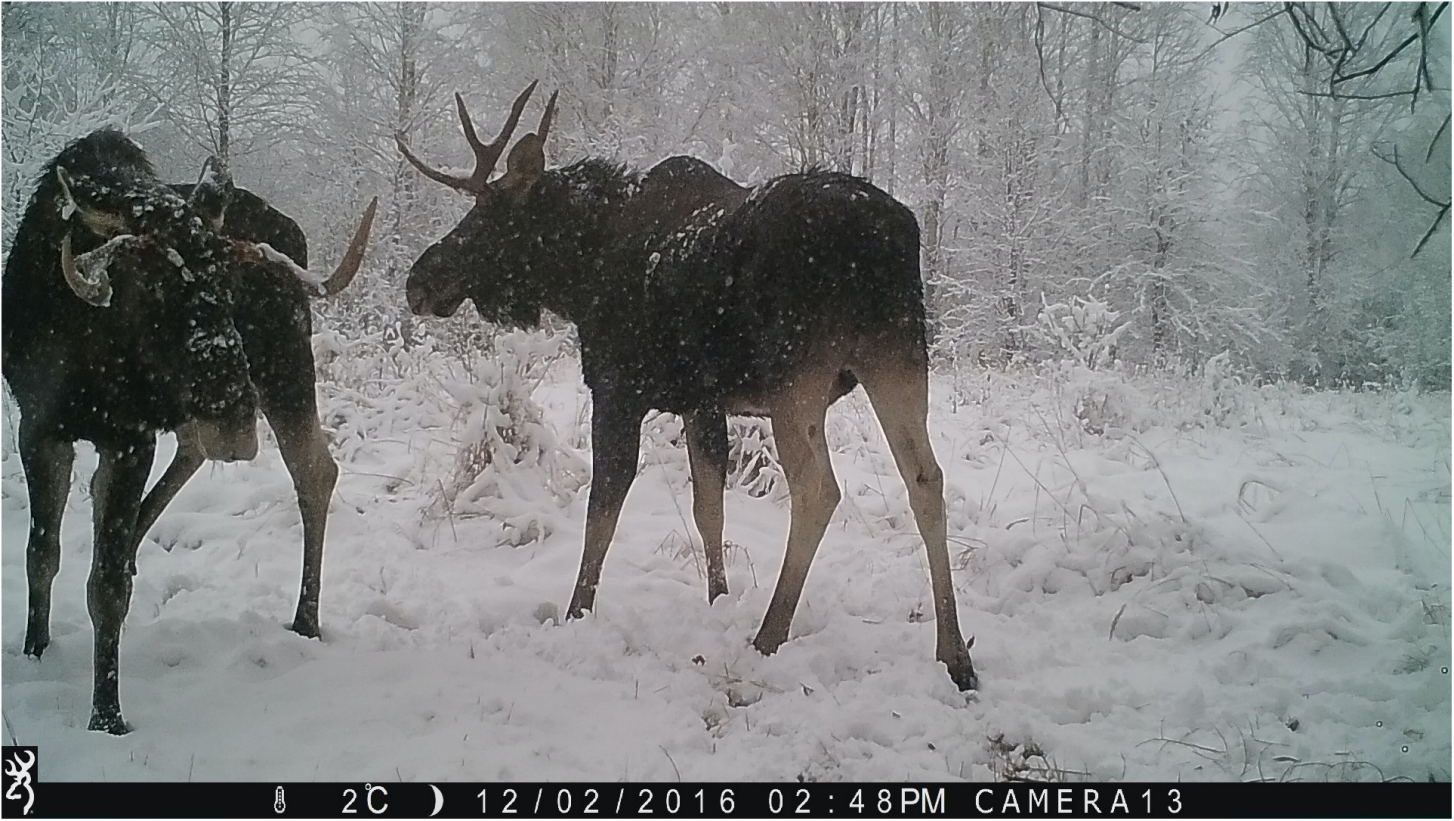
Sample photograph of precipitation taken from a camera trap in Chernobyl. Species shown are moose (*Alces alces*).

**FIGURE 6:**
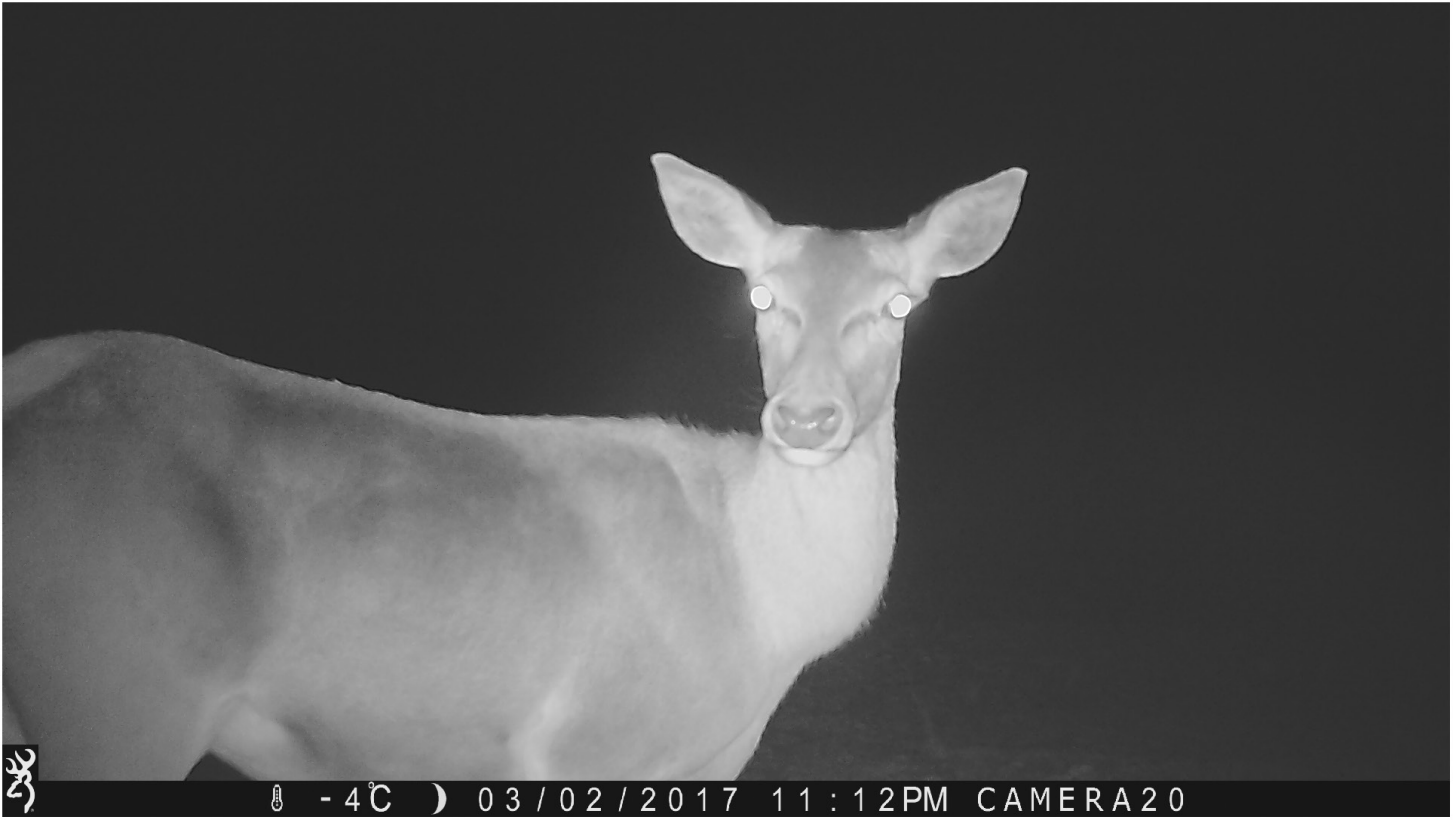
Sample photograph of the camera traps deploying infrared LED technology, signifying low light levels. Species shown is a red deer (*Cervus elaphus*).

After generating the case study data, we ran a generalized linear mixed model (GLMM) on R version 4.2.2 with the package “lme4” to tease apart the effects of low light/day, precipitation/no, cloud cover, temperature, wind speed, and image contrast on CNN success. We chose to run a GLMM due to their ability to build on simple linear regression by capturing complex relationships with fixed covariates and factors, including random effects (Bono et al., 2021). Prior to constructing the GLMM model, all continuous predictors were centered, standardized, and checked for high Pearson correlation to remove multicollinear variables. Given contrast and night vs day has a significant correlation (0.82), the predictor night vs day was removed to keep the more descriptive predictor. The predictor temperature contained outliers which are outside the IQR range that resulted in only four events being removed from the training set of the GLMM. Low light/day and precipitation/no were dummy variables—we were unable to make these variables continuous due to the difficulty involved in quantifying amount of light; quantifying precipitation was also not reliable due to its transient nature. Historical weather forecasts may state that it rained a certain amount on a given day or hour, but this did not necessarily correspond to actual precipitation present in the images due to the camera's specific location. For example, it may have rained 2 inches in a general location over a certain period of time, but it may not have been raining at the exact moment in time at the specific location where the picture was taken. Therefore, the presence or absence of precipitation in each image was noted visually (see Figure 5). Amount of daylight (low light vs. day) was determined based on whether or not the camera trap deployed the use of infrared LEDs, signifying low light levels, that is, night (see Figure 6). While the amount of light in an area can in general be determined based on time of year and location, we used this conservative approach to determining day vs. night because it avoids confounding variables such as cloud cover, camera orientation, and shadows. Cloud cover, temperature, wind speed, and contrast were continuous variables. These meteorological data were obtained either from World Weather Online or the image itself, as in the case of temperature (World Weather Online, 2022; each camera recorded ambient temperature along with date and time of day). Overall image contrast was determined by running the case study images through the R package imagefluency.

## 3 RESULTS

### 3.1 Raw data

To compile our train, test, and validation sets, we observed 14 animal species in a total of 12,157 camera trap images taken from 45 locations across the CEZ. To analyze the effects of ambient conditions on CNN success, we used an unlabeled image set for our case study consisting of 9576 new images (see Table 2). Maps of these camera trap locations are shown in Figures 2 and 4. Frequency of animal detections, both CNN and human‐classified are shown in Table 3.

**TABLE 2:** Description of the train, test, validation, and case study data sets.

Dataset	Description
Train	Set of 3620 images used to define, or “train,” the CNN architecture. See Figure 2.
Test	Set of 402 images used to evaluate, or “test,” the trained CNN's performance after each iteration of the model construction. See Figure 2.
Validation	Set of 8135 images used to extrapolate trained architecture to a different set of images. See Figure 2.
Case study	Set of 9576 images used to assess influence of ambient settings on CNN success. See Figure 4.

**TABLE 3:** Number of each species identified by humans (actual) vs. number of each species the CNN identified (predicted) from images taken from 12 randomly selected cameras within the case study.

Species	Actual	Predicted
Boar	7	8
Fox	7	3
Hare	15	22
Horse	25	25
Moose	25	29
Raccoon	2	2
Red deer	93	128
Roe deer	7	13
Wolf	1	2
Totals	182	232

### 3.2 Training metrics

After training, the CNN's predicted values and ground truths were summarized in a confusion matrix (see Appendix S1). Based on the number of false positives, false negatives, true positives, and true negatives the following metrics outlining model performance were calculated: accuracy = 81.31, precision = 97.93, recall = 78.56, and F − 1 = 87.18. These metrics were calculated at a confidence threshold of 0.9.

### 3.3 CNN case study

Human and CNN predictions were generated for nine of the most prevalent animal species—which consisted mainly of relatively large mammals—present in 114 events across 9576 images taken from 12 locations across the CEZ (see Table 3).

**FIGURE 7:**
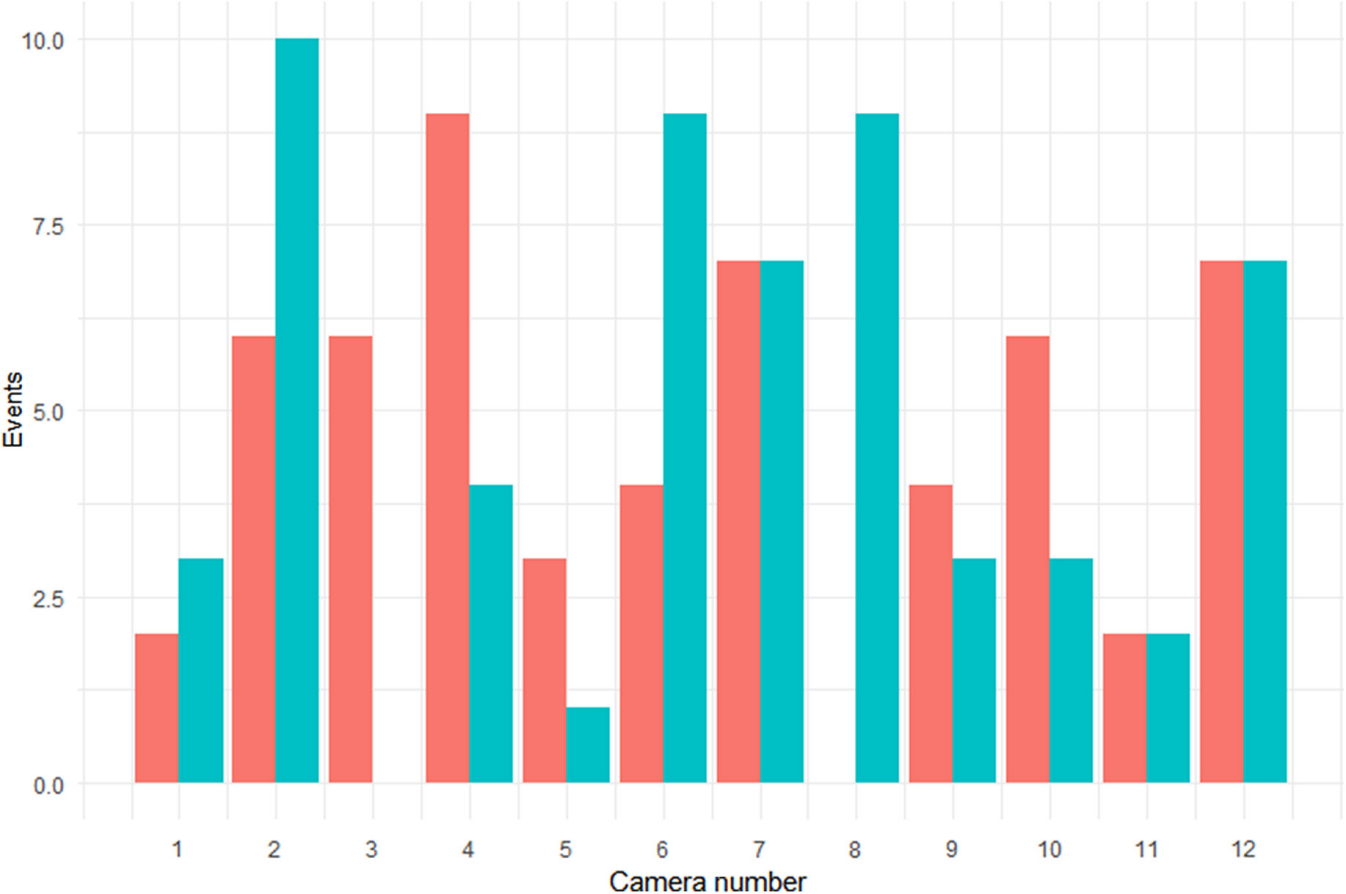
Number of CNN successes and failures per camera, which together constitute total events. Failures are shown in red, and successes are shown in blue. See Table 3.1 in Appendix S3 to view how camera numbers 1–12 correspond to camera names.

A total of 114 events were classified across 12 different cameras. Seven cameras were at least 50% successful (Figure 7). The total count predicted by the CNN exceeded the count identified by humans, delineating a significant number of false positives (Table 3).

### 3.4 GLMM model selection and evaluation

A generalized linear mixed‐effects model (GLMM) with binary response was constructed with the popular “lme4” package. The fixed factors were binary categories consisting of no precipitation/precipitation and night/day (0/1). The fixed covariates were temperature (°C), windspeed (km/h), and contrast. This allows us to see how these predictors and to what degree each predictor has a relationship with the response of successful CNN classification. The random effects are the cameras capturing images for the events, thus accounting for the hierarchical clustering in model construction. Finally, a Pearson correlation analysis was run to remove multicollinearity (see Table 4). Night/day was removed due to the high positive correlation with contrast and a small positive correlation with precipitation to retrain the predictors with finer measurements of image clarity and differentiation, including fog or precipitation effects. This study followed the statistical convention of comparisons across a nested model structure to a null (see Table 5). Predictors included in each model are outlined in Table 6.

**TABLE 4:** Pearson correlation matrix summarizing the model parameters, AIC, BIC, log‐likelihood, deviance, degrees of freedom, and *p*‐value following the nested model comparison using analysis of variance (ANOVA).

	Contrast	Temperature	Wind speed	Cloud cover	Night/day	Precipitation/no
Contrast	1	0.03	0.19	0.06	0.87	0.23
Temperature	0.03	1	0.22	−0.09	0.06	0.04
Wind speed	0.19	0.22	1	0.24	0.10	0.04
Cloud cover	0.06	−0.09	0.24	1	0.01	−0.22
Night/day	0.87	0.06	0.10	0.01	1	0.33
Precipitation/no	0.23	0.04	0.04	−0.22	0.33	1

**TABLE 5:** GLMM outputs demonstrating that the model does not perform better than random chance (null), that is ambient predictors do not linearly affect CNN success.

Model	AIC	BIC	logLik	Deviance	Chisq	Df	Pr (>Chisq)
Null	152.54	157.94	−74.270	148.54			
2	154.25	162.35	−74.124	148.25	0.2923	1	0.58878
3	155.14	165.94	−73.570	147.14	1.1086	1	0.29239
4	154.32	167.82	−72.159	144.32	2.8207	1	0.09306
5	153.31	169.51	−70.654	141.31	3.0101	1	0.08275
1	155.06	173.96	−70.531	141.06	0.2472	1	0.61902

**TABLE 6:** The null and models 1–5 within the GLMM and their corresponding parameters.

Model number	Equation representation
Null	CNN_success ~1 + (1 | camera_number)
1	CNN_success ~ contrast + windspeed + precipitation + temperature + cloud_cover + (1 | camera_number)
2	CNN_success ~ contrast + (1 | camera_number)
3	CNN_success ~ contrast + windspeed + (1 | camera_number)
4	CNN_success ~ contrast + windspeed + precipitation + (1 | camera_number)
5	CNN success ~ contrast + windspeed + precipitation + temperature + (1 | camera_number)

*Note*: Predictors analyzed include contrast, wind speed, precipitation, temperature, and cloud cover. Starting from model 2 with contrast, each subsequent model incorporates another predictor. Model 1 includes all predictors.

Upon analyzing wind speed, temperature, precipitation, contrast, and cloud cover via GLMM, no strong significant linear relationship between these variables and CNN success was found with the exception of temperature (see Table 5). Additionally, as shown by Model 4, precipitation demonstrates some possible improvement of the GLMM. However, if we only compare models 4 and 5 to our null, model 4 is no longer significantly improving model performance. Thus, significance here is a by‐factor of other predictors not correlating with CNN performance (see Table 4.1 in Appendix S4).

Realizing the assumptions of the poorly fitted model, more predictors must be collected to better capture the relationship of CNN success and environmental predictors, but temperature does improve model fit in all scenarios of model construction. Furthermore, upon performing a Point Biserial correlation between temperature and CNN success rate a visual positive relationship was shown (see Figure 4.2 in Appendix S4).

Based on AIC values, we can assess how well the addition of covariates explains Kullback–Leibler (KL) divergence compared to the null. Model 5 has the lowest AIC value to explain KL divergence and is statistically significantly (*p*‐value < .05) over simpler models. However, significance does not improve over a null model in direct ANOVA comparison, thus GLMM improvement is a by‐factor of previous predictors not correlating with CNN performance.

Upon reading the model diagnostics, the residuals of the deviance appear to follow a relationship with the fitted values of the model. Thus, conclusions from this model alone must be made carefully as the relationship is non‐linear and/or does not contain all predictors of CNN success. The residual v. fitted values plot (see Figure 4.3 in Appendix S4) demonstrates that this is not a linear relationship, and more values would be necessary to determine whether there is a non‐linear relationship at play.

### 3.5 Light level and precipitation

After counting successes and failures in the presence or absence of precipitation, the CNN performed better when precipitation was not present; the success rate in clear weather was 21.99% higher than the success rate when it was raining or snowing (Table 7). Furthermore, after assessing successes and failures in low light/daytime conditions, the CNN was 13.11% more successful during the day than during low light conditions (Table 8). As noted previously, these findings correlate with the suggestion that there is some sort of relationship between precipitation and CNN success, but it is not a linear relationship as determined by the construction of the GLMM.

**TABLE 7:** Number of successes and failures by the CNN in the presence/absence of precipitation.

Precipitation	Successes	Failures	Success rate
Present	10	19	34.48%
Not present	48	37	56.47%

*Note*: An event occurred in the presence of precipitation if there was visual snowfall or rainfall noted, all other events occurred in the absence of precipitation.

**TABLE 8:** Number of successes and failures by the CNN in low light vs. daytime conditions.

Time of day	Successes	Failures	Success rate
Low light	31	37	45.59%
Day	27	19	58.70%

*Note*: Low light conditions were delineated by the deployment of infrared LEDs; images that did not deploy the LEDs were classified as taken during the daytime.

## 4 DISCUSSION

Overall, these findings suggest that there is no significant linear relationship between ambient conditions and CNN success when the CNN is trained with a dynamic image set consisting of pictures taken in a wide variety of meteorological conditions. There could potentially be a more complex, non‐linear relationship between predictors and CNN success, but more sampling power would be necessary to run this analysis.

In regard to temperature, while we did not find that it definitively had a causative effect on CNN success, higher temperatures were shown to have a relationship with the CNN success rate. This is likely because temperatures below freezing are associated with frozen precipitation, especially snowfall. Precipitation was also shown to have a negative association with CNN success (see Table 7), and low light levels were also associated with CNN failure (see Table 8). In the presence of frozen precipitation and in the absence of sufficient light, CNNs may be less successful at image classification due to low levels of contrast between the object and its background (Tao et al., 2017) which would create a relatively blurred object contour. However, no significant linear correlation between CNN success and precipitation/low light levels was found due to training the model with a large number of images taken when temperatures were below freezing, which improves the CNN for cold‐weather image classification.

We expected that image contrast would play a major role in CNN accuracy. High contrast levels are necessary for effective image classification because the CNN is expected to be better able to distinguish indistinct targets from cluttered backgrounds (Fan et al., 2018). However, we found that the overall contrast of our images did not have an effect on CNN success, which suggests that the issue is not necessarily contrast as a whole on a given image but rather could be attributed to the definition of object contours.

The lack of a relationship between wind speed and CNN success is interesting as we expected that high winds would negatively impact CNN success rates on account of moving vegetation triggering the camera (Glen et al., 2013; Zhang et al., 2016). Certainly, it has been frequently observed that wind effects on vegetation can produce massive increases in the generation of images not containing any animal targets. Vegetation triggering can be problematic for CNNs in that the model has to sort a large number of true negatives that may be incorrectly classified as false positives—thus reducing overall accuracy. However, by training the CNN with images taken in a variety of meteorological conditions, including high winds, we were able to account for factors such as these.

This study suggests that ambient meteorological conditions can be accounted for if the CNN is trained with a large and dynamic image set. The training set should include each target species in a variety of orientations and weather conditions. As a means to further mitigate the effects of meteorology and its subsequent effects on image contrast, we suggest cropping the animal from its background to enhance image classification accuracy (Beery et al., 2019; Yu et al., 2013) although this adds to the manual labor required for the process.

Future camera trap studies may be further enhanced by performing preliminary pilot studies to determine which camera model best meets the requirements of the study (Newey et al., 2015). The optimal camera model can depend on a variety of variables, such as target species, site accessibility, habitat, and climate (Rovero et al., 2013). Also, there is tremendous variation among camera makes and models in their resolution, field of view and low light capabilities. Higher quality cameras may produce dramatically better images and be far more durable under field conditions than cheaper consumer‐grade cameras, but this comes at much greater cost which is an important consideration given the need for large numbers of cameras (hundreds) for ecological studies. Consumer‐grade cameras offer the great advantage of being almost disposable. In our study, we used the relatively inexpensive Browning Recon Force FHD trail camera. While these cameras are not high‐end, we chose to use them because our research focuses on how small labs with limited resources can utilize CNNs in conjunction with camera traps. Furthermore, Browning cameras are designed and manufactured by hunters and thus the PIR sensors are tailored toward deer‐sized animals. The majority of species we looked at (boar, horses, moose, red deer, roe deer, and wolves) are relatively large mammals (see Table 3) and thus produce a heat signature similar to a deer and are readily picked up by the PIR sensor (Wearn & Glover‐Kapfer, 2017). However, it should be noted that if our target species were relatively small (<1 kg), we would recommend using a more sensitive camera model. In addition, camera model may be particularly important for night/low light conditions such as at dawn, dusk, or during daytime precipitation (Rovero et al., 2013), especially given that many of the animal targets are particularly active at night. To our knowledge, there have been few comparative studies of camera performance under varying environmental conditions.

There were several limitations to the present study, most importantly the use of a relatively small sample size of 114 unique events for CNN training. This constraint was dictated primarily by both human time and effort restrictions and the need for greater computational power. Future studies should analyze a larger number of events, in addition to analyzing background clutter or object contours as variables that may influence CNN success. Although manpower will always be in short supply, desktop computational power continues to rise exponentially thus providing the opportunity for enhanced CNN development. We were able to train an effective CNN that accounts for ambient conditions using limited computational power and a small image set.

Classification models that use CNNs are becoming increasingly useful for the processing of camera trap imagery (Norouzzadeh et al., 2018). While CNNs can be time and cost effective, it is difficult to achieve the accuracy levels provided by manual (i.e., human) analyses (Favorskaya & Pakhirka, 2019). However, characterization of the ecological and environmental characteristics of the study site and the use of a dynamic image training set, can greatly enhance the utility of artificial intelligence (AI) tools like CNNs.

Finally, on a more general note, camera traps have become an increasingly important tool for the monitoring of vertebrates, both because they are cost effective and relatively easy to deploy, but especially because many animals are extremely difficult to monitor at landscape scales using any other method. This tool is useful for monitoring shy and rare species that are in hard‐to‐reach locations either because of geography or because of military conflict, as is currently the case in Ukraine. Our studies of mammals in the Chernobyl Exclusion Zone have continued despite the ongoing conflict because the cameras are semi‐autonomous and can be left in place for extended periods (several months) without human intervention. The development of automated image processing will greatly facilitate data processing, and the generation of accurate and precise datasets will continue to depend on enhancements of camera design and the incorporation of independent variations (e.g., meteorological conditions) into the training image set.

## AUTHOR CONTRIBUTIONS

**Rachel E. Maile:** Conceptualization (lead); data curation (equal); formal analysis (equal); funding acquisition (supporting); investigation (lead); methodology (equal); project administration (lead); resources (supporting); software (supporting); supervision (supporting); validation (lead); visualization (lead); writing – original draft (lead); writing – review and editing (equal). **Matthew T. Duggan:** Conceptualization (supporting); data curation (equal); formal analysis (equal); investigation (equal); methodology (equal); project administration (supporting); software (lead); supervision (supporting); validation (equal); visualization (equal); writing – original draft (supporting); writing – review and editing (equal). **Timothy A. Mousseau:** Conceptualization (lead); data curation (equal); formal analysis (equal); funding acquisition (lead); investigation (supporting); methodology (lead); project administration (lead); resources (lead); software (supporting); supervision (equal); validation (supporting); visualization (supporting); writing – original draft (supporting); writing – review and editing (supporting).

## FUNDING INFORMATION

Funding was provided by the Samuel Freeman Charitable Trust and the University of South Carolina Honors College.

## CONFLICT OF INTEREST STATEMENT

The authors declare no conflicts of interest.

## Supporting information


Appendix S1:

## Data Availability

The data that support the findings of this study are available in BioStudies at https://www.ebi.ac.uk/biostudies/studies/S‐BSST1171, reference number S‐BSST1171.
