# Supplementary material for: The successes and pitfalls: Deep‐learning effectiveness in a Chernobyl field camera trap application
Source: Ecol Evol. 2023 Sep 5;13(9):e10454. doi: 10.1002/ece3.10454 (PMC10477951; doi:10.1002/ece3.10454)
Supplement: Supplementary file 1 — Appendix S1: [file ECE3-13-e10454-s001.zip › ece310454-sup-0005-AppendixS1.docx]

**Appendix**

Appendix 1

**SI Table 1.1**: Confusion matrix (CM) of ground truth vs. predicted values for 14 object classes at a confidence threshold of 0.9. The accuracy, precision, recall, and F1 scores (as determined by the true negatives, true positives, false positives, and false negatives shown in the CM) are 81, 98, 79, and 87, respectively.

[chernobyl confusion matrix](https://docs.google.com/spreadsheets/d/1DkTDfNh8XF8d9cjuHOrtW_QscjNVcwVLDTSiN2nfsiM/edit?usp=sharing)

Appendix 2

**SI Figure 2.1**: Images showing site-specific factors of the 12 case study camera traps. Images were taken on the day the cameras were set up. Reading left to right, row one: CH02B, CH11B, CH12; row two: CH13, CH15, CH16B; row three: CH17B, CH18, CH19; row four: CH20, CH21B, CH22.

**SI Figure 2.2**: Image demonstrating general camera orientation and height at 1.22 meters above the ground.

Appendix 3

**SI Table 3.1:** Camera names that correspond to each camera number.

| **Camera Number** | **Camera Name** |
| --- | --- |
| 1 | CH02B |
| 2 | CH11B |
| 3 | CH12 |
| 4 | CH13 |
| 5 | CH15 |
| 6 | CH16B |
| 7 | CH17B |
| 8 | CH18 |
| 9 | CH19 |
| 10 | CH20 |
| 11 | CH21B |
| 12 | CH22 |

Appendix 4

**SI Table 4.1**: Comparisons of solely the null vs. Model 4 (CNN_success ~ contrast + windspeed + precipitation + (1 | camera_number) and solely the null vs. Model 5 (CNN success ~ contrast + windspeed + precipitation + temperature + (1 | camera_number).

| **Model** | **AIC** | **BIC** | **logLik** | **Deviance** | **Chisq** | **Df** | **Pr(>Chisq)** |
| --- | --- | --- | --- | --- | --- | --- | --- |
| Null | 152.54 | 157.94 | -74.270 | 148.54 |  |  |  |
| 4 | 154.32 | 167.82 | -72.159 | 144.32 | 4.2216 | 3 | 0.2385 |

| **Model** | **AIC** | **BIC** | **logLik** | **Deviance** | **Chisq** | **Df** | **Pr(>Chisq)** |
| --- | --- | --- | --- | --- | --- | --- | --- |
| Null | 152.54 | 157.94 | -74.270 | 148.54 |  |  |  |
| 5 | 153.31 | 169.51 | -70.654 | 141.31 | 7.2316 | 4 | 0.1241 |

**SI Figure 4.2**: Linear regression model of temperature versus success rate (y = 0.0219x + 0.637; *R²* = 0.294). Point Biserial Correlation Coefficient suggests a relationship between temperature and success rate (*r_pb_* = 0.2325; *p* value = 0.0128).

**SI Figure 4.3**: Deviance residuals vs. fitted values plot demonstrating that the relationship between ambient conditions and CNN success is non-linear.

**SI Figure 4.4**: Q-Q plot demonstrating model diagnostics, i.e. the data is normally distributed.
