# Supplementary figures and images for: The successes and pitfalls: Deep‐learning effectiveness in a Chernobyl field camera trap application
Source: Ecol Evol. 2023 Sep 5;13(9):e10454. doi: 10.1002/ece3.10454 (PMC10477951; doi:10.1002/ece3.10454)

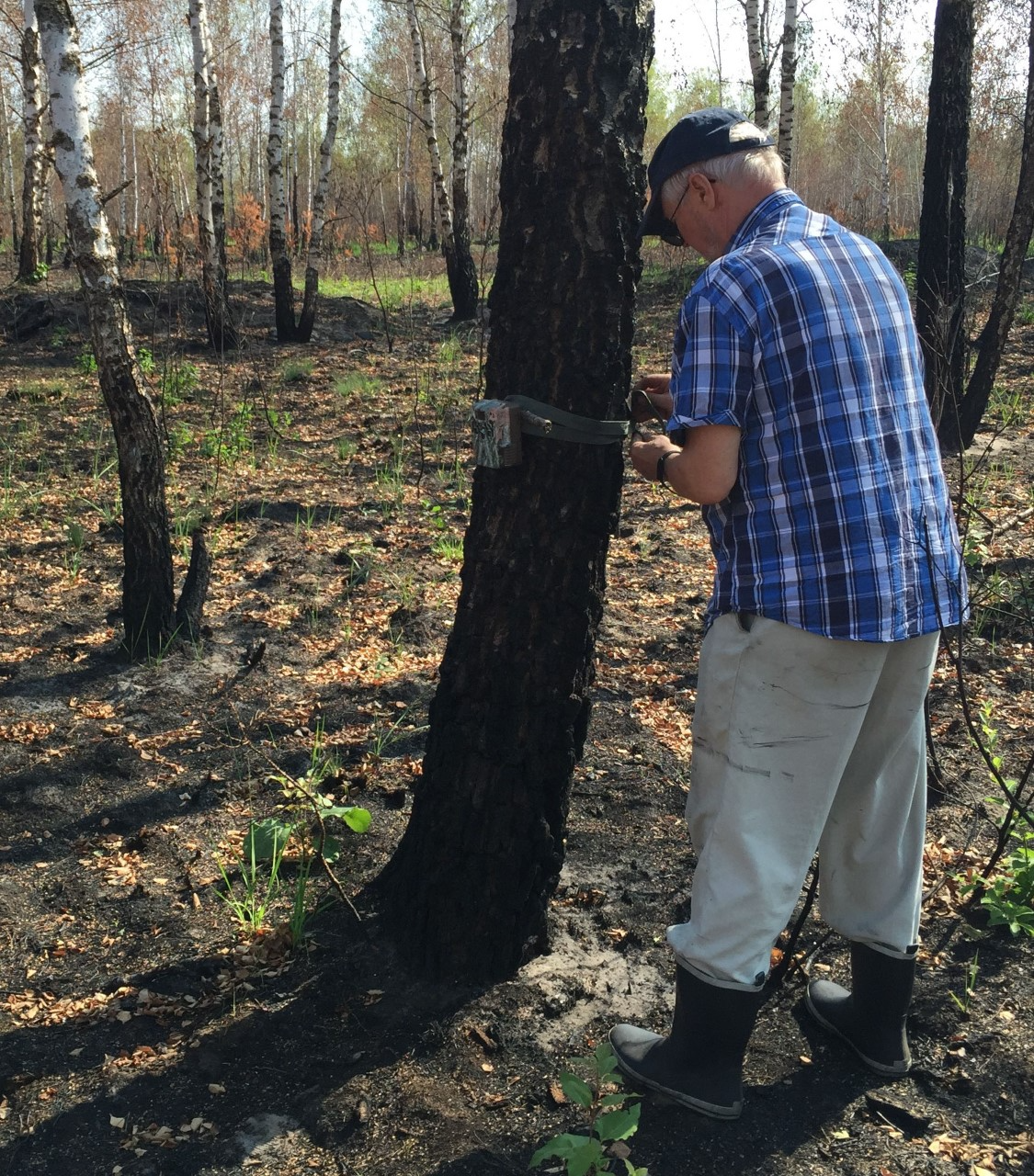

Supplement: Supplementary file 1 — Appendix S1: [file ECE3-13-e10454-s001.zip › appendixfigure2.2.png]

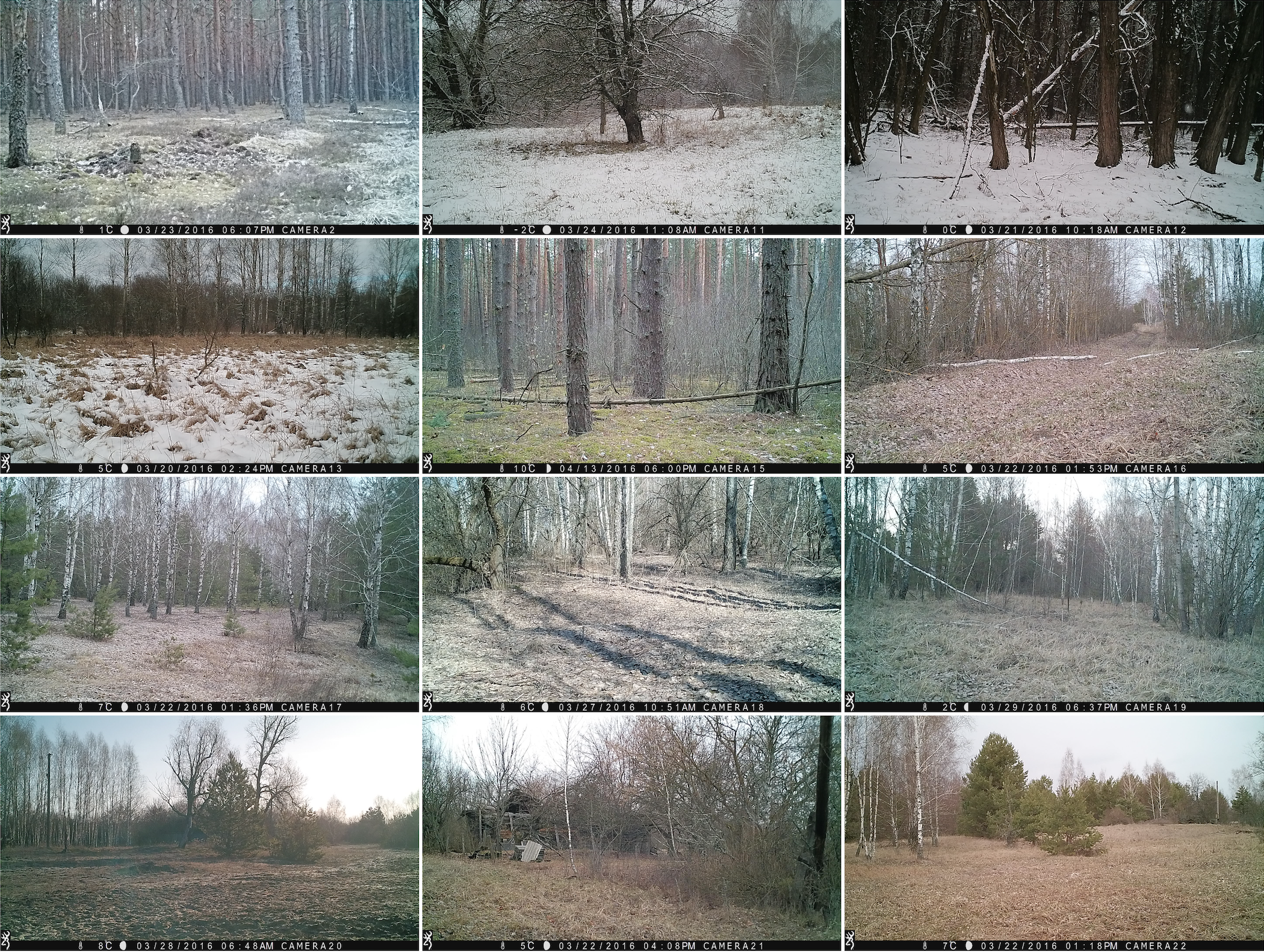

Supplement: Supplementary file 1 — Appendix S1: [file ECE3-13-e10454-s001.zip › ece310454-sup-0001-FigureS1.png]

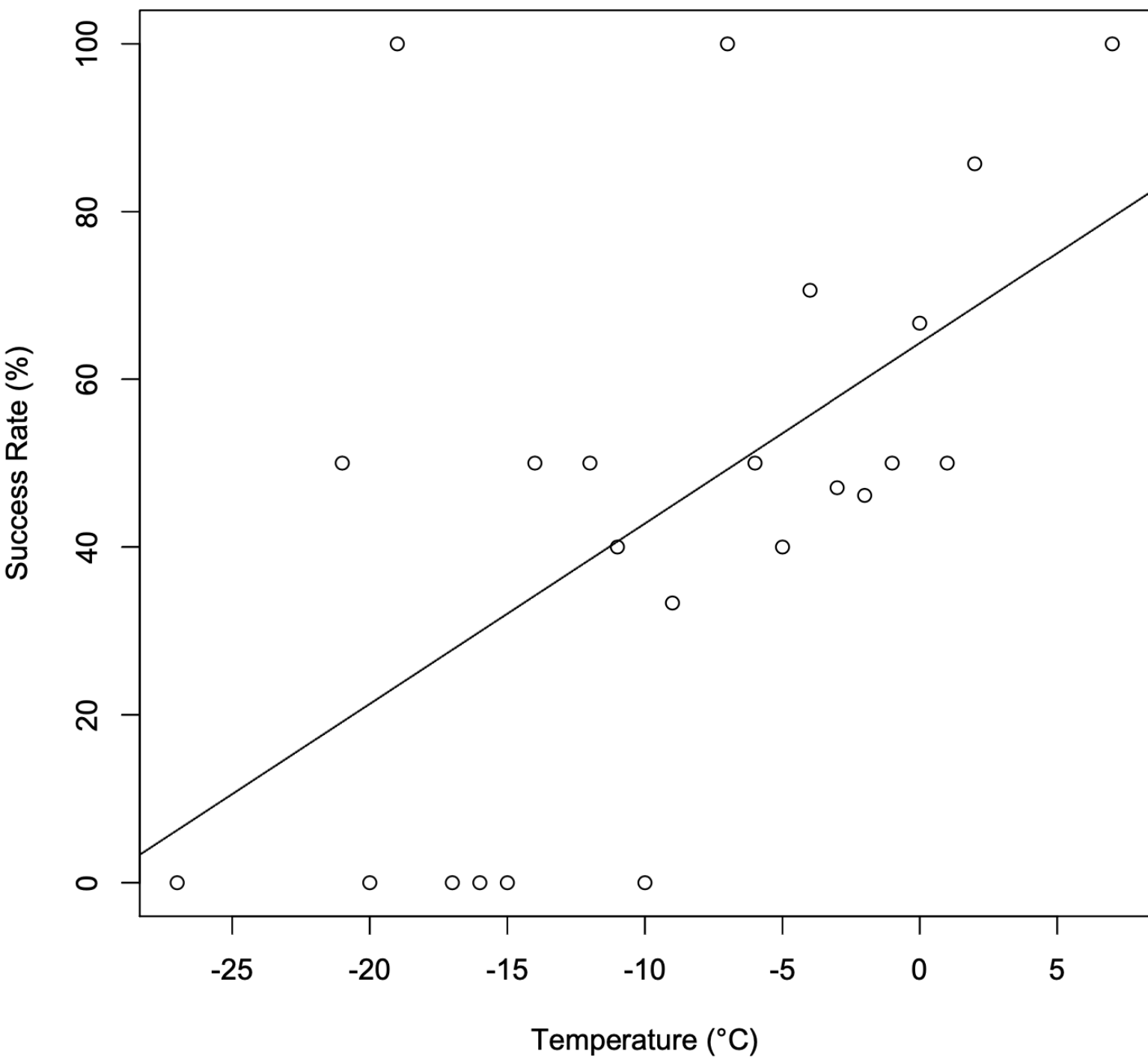

Supplement: Supplementary file 1 — Appendix S1: [file ECE3-13-e10454-s001.zip › ece310454-sup-0002-FigureS2.pdf]

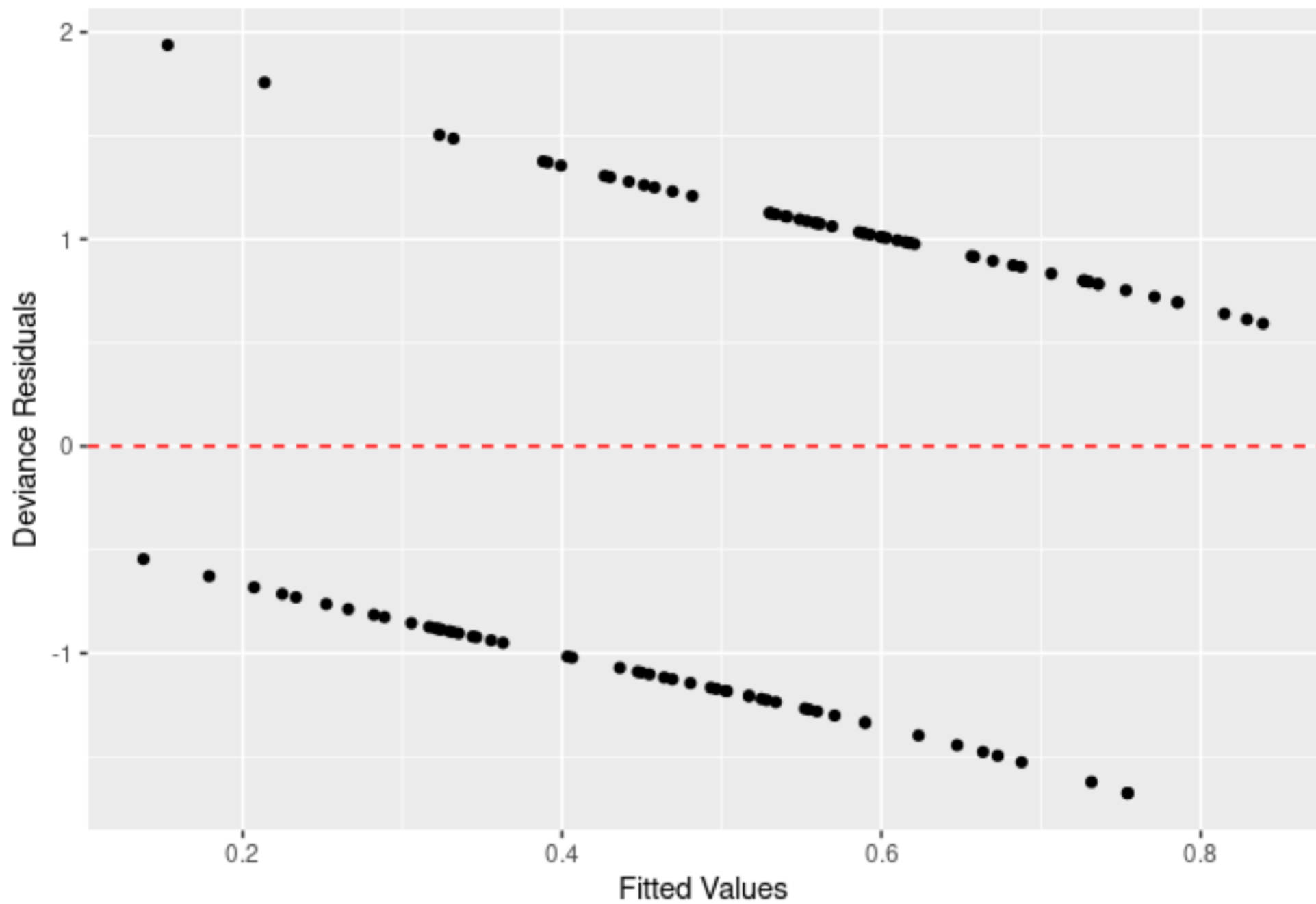

Supplement: Supplementary file 1 — Appendix S1: [file ECE3-13-e10454-s001.zip › ece310454-sup-0003-FigureS3.pdf]

Normal Q-Q Plot

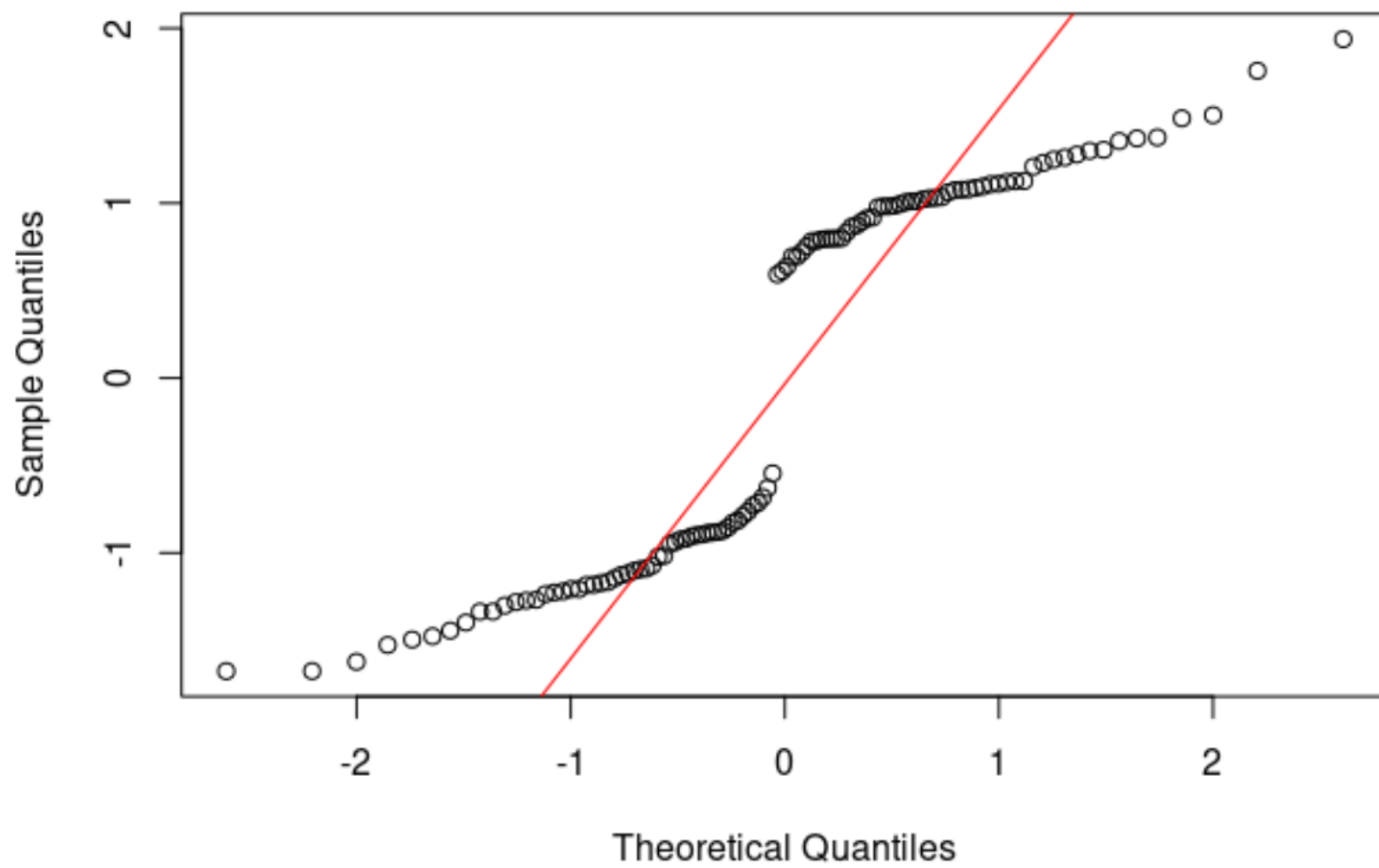

Supplement: Supplementary file 1 — Appendix S1: [file ECE3-13-e10454-s001.zip › ece310454-sup-0004-FigureS4.pdf]
